# Guided to Success: Optimizing Implant Positioning With Global Positioning System (GPS) Navigation in Total Shoulder Arthroplasty

**DOI:** 10.7759/cureus.101816

**Published:** 2026-01-18

**Authors:** Victor L Tran, Katie E Zhang, David J Novak, Jami Fano

**Affiliations:** 1 Research, Edward Via College of Osteopathic Medicine, Blacksburg, USA; 2 Research, Case Western Reserve University School of Medicine, Cleveland, USA; 3 Orthopedic Surgery, OrthoVirginia, Fair Oaks, USA

**Keywords:** anatomic total shoulder arthroplasty, gps navigation, implant alignment, orthopedic navigation, real-time computer-assisted surgery, shoulder osteoarthritis

## Abstract

We present the case of a 48-year-old male with severe right glenohumeral osteoarthritis who experienced progressive pain and functional loss despite extensive conservative management. Given his high activity level and preserved rotator cuff, he underwent a global positioning system (GPS)-navigated anatomic total shoulder arthroplasty (aTSA) using the ExactechGPS system (Exactech, Inc., Gainesville, FL). The procedure was uncomplicated, and postoperative rehabilitation progressed without limitation. Postoperative imaging demonstrated accurate component positioning with correction of posterior humeral head subluxation. This case highlights the short-term clinical and radiographic outcomes following navigation-assisted aTSA in a younger, active patient.

## Introduction

Degenerative joint disease in younger patients presents a significant challenge in orthopedic surgery, as few interventions provide both durable outcomes and preservation of function. In the shoulder, glenohumeral osteoarthritis is commonly managed with nonoperative measures such as physical therapy, nonsteroidal anti-inflammatory drugs, and corticosteroid injections. While these modalities can offer temporary relief, they do not alter the course of disease progression. For definitive resolution of symptoms and joint-spacing pathology, anatomic total shoulder arthroplasty (aTSA) remains the gold standard in patients with an intact rotator cuff. In the United States, the overall incidence of shoulder replacement has increased more than threefold since the early 2000s, now exceeding 200,000 procedures annually [1]. The longevity of aTSA in younger, active patients remains a challenge; prosthetic wear and glenoid loosening continue to limit implant survival.

Historically, aTSA has been performed predominantly in older, lower-demand patients, with the mean age at surgery ranging from 65 to 70 years [2]. Only 3%-7% of all aTSAs are performed in patients younger than 50, most commonly in the context of post-traumatic arthritis, avascular necrosis, or inflammatory disease rather than primary degenerative osteoarthritis [2]. Younger Individuals pose a unique challenge due to their higher activity levels, longer life expectancy, and increased risk of glenoid component wear or loosening over time, the most common cause of prosthesis failure. Accurate glenoid positioning is a critical factor influencing long-term outcomes and implant longevity, and navigation has been shown to reduce outlier rates in component alignment compared with conventional techniques [3]. 

We present the case of a 48-year-old male who underwent an aTSA with the ExactechGPS navigation system (Exactech, Inc., Gainesville, FL) for end-stage degenerative osteoarthritis of the right glenohumeral joint. The patient, an active individual who coaches youth baseball, referees wrestling matches, and regularly engages in weight training and hunting, had previously completed extensive conservative management, including physical therapy and three glenohumeral corticosteroid injections. Although the injections provided several months of relief, his pain and stiffness progressively recurred, limiting both daily function and recreational activities. Given his intact rotator cuff and desire to maintain an active lifestyle, an aTSA with ExactechGPS navigation system (Exactech Inc.) was chosen to maximize component alignment and long-term implant survival.

## Case presentation

The patient was a 48-year-old male who presented to our orthopedic clinic in December 2022 for evaluation of right shoulder pain that began 1.5 years earlier, in the summer of 2021, while playing baseball. He had a past medical history of gastroesophageal reflux disease (GERD) but no other comorbidities contributing to his shoulder pain. The patient was a highly active person and was participating in baseball, wrestling, hunting, and Spartan racing at the onset of his symptoms. He reported being unable to pitch overhead in baseball and unable to raise his arm without pain.

**Figure 1:**
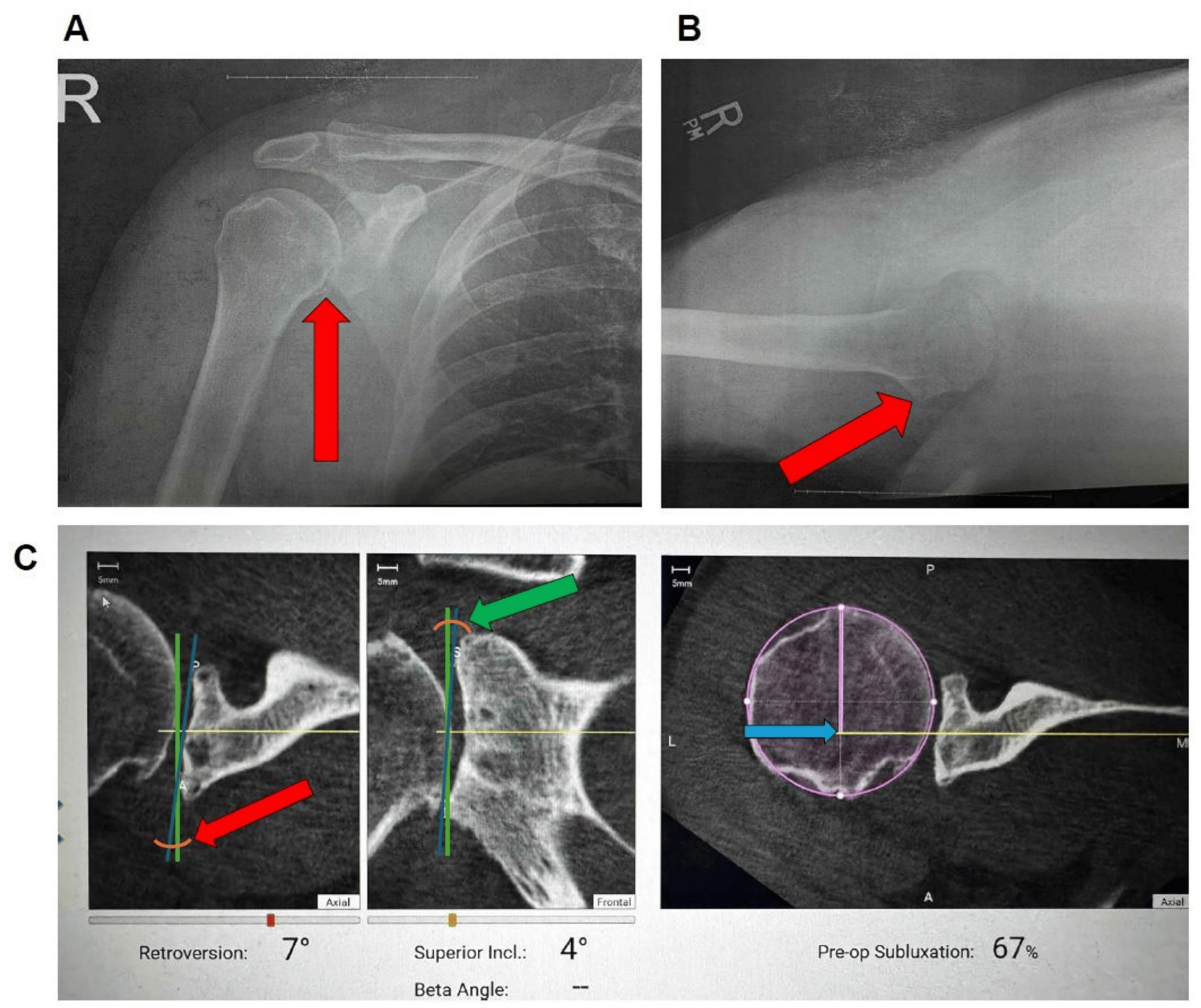
Preoperative imaging and ExactechGPS navigation system (Exactech, Inc., Gainesville, FL) planning for anatomic total shoulder arthroplasty. (A) Anteroposterior radiograph of the right shoulder demonstrating inferior humeral head osteophyte formation with associated subchondral sclerosis, consistent with early glenohumeral osteoarthritis. (B) Axillary radiograph of the right shoulder demonstrating humeral head contour irregularity, consistent with degenerative change. (C) Preoperative GPS planning images quantifying glenoid retroversion of 7° (red arrow), superior inclination of 4° (green arrow), and posterior humeral head subluxation of 67% (blue arrow), consistent with eccentric posterior wear. GPS, global positioning system

Physical examination of the right shoulder at the patient’s initial visit was notable for 60° of external rotation with tightness, forward flexion to 170°, 4/5 external rotation strength, and 0/5 supraspinatus strength. He had a positive drop arm test. X-rays taken at the visit showed bone spurring and narrowing of the glenohumeral joint space. Given these findings, the patient was referred for an MRI, which showed severe glenohumeral osteoarthritis with glenoid labral degeneration, posterior subluxation of the humeral head, and posterior paralabral cyst formation. Preoperative radiographs demonstrated advanced glenohumeral osteoarthritis (Figure 1).

Additionally, supraspinatus tendinosis and focal intrasubstance tendon was tearing at the tendon footprint without evidence of a full-thickness rotator cuff tear.

Given the MRI findings and symptoms, a total shoulder arthroplasty was advised as the definitive treatment for the patient’s right shoulder pain. However, the patient elected to hold off on surgery until he was finished with his commitments as a wrestling referee and after his South African hunting safari at the end of 2023. During this period of about 14 months, he received three cortisone injections into the right shoulder, which provided relief of his symptoms for several months but with limited effectiveness by the summer of 2023. The patient reported increased pain when performing activities of daily living, including driving and working out in the gym.

Ultimately, the patient underwent an aTSA with the ExactechGPS navigation system (Exactech, Inc.) due to increased complexity for tunnel and glenoid cap placement in February 2024. The patient was an ideal candidate for this procedure as he was a healthy, young, and athletic individual.

**Figure 2:**
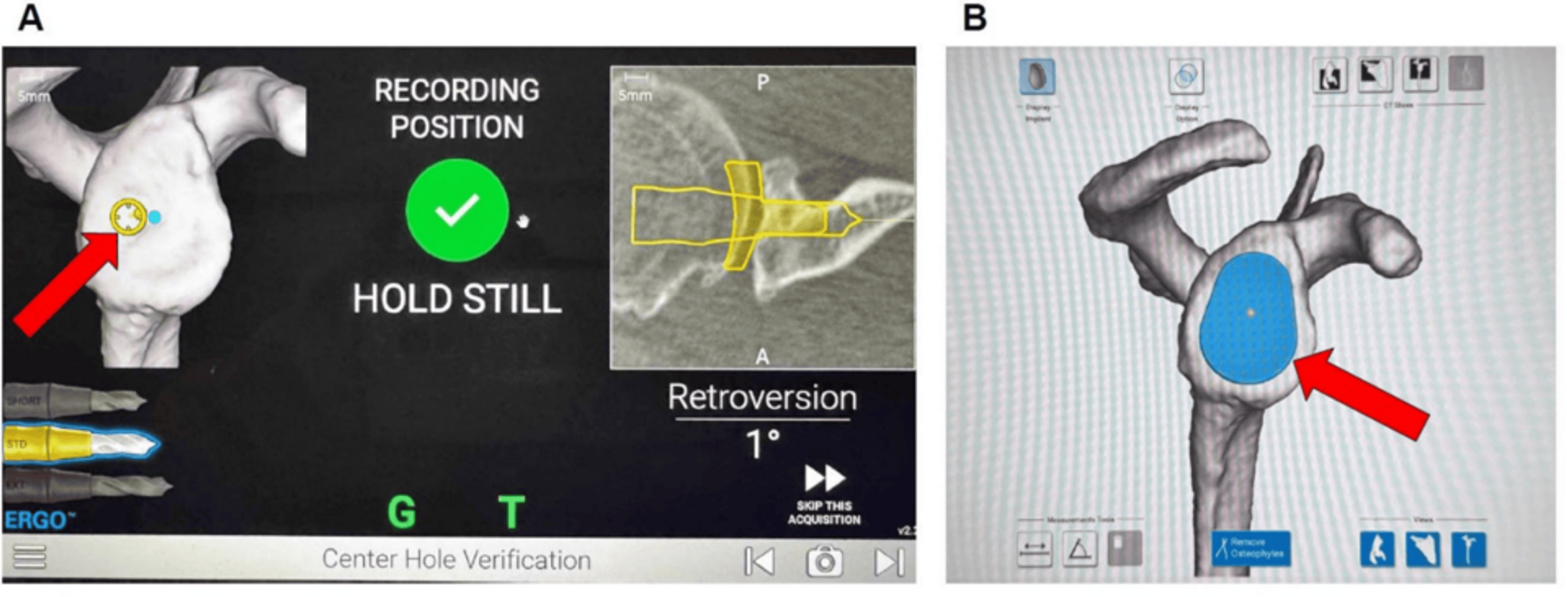
Intraoperative ExactechGPS navigation system (Exactech, Inc., Gainesville, FL) during anatomic total shoulder arthroplasty. (A) Real-time intraoperative verification confirming accurate digital central guide-pin targeting within 1° of the planned retroversion and 4° superior inclination at the central glenoid guide-pin target (red arrow). (B) Three-dimensional reconstruction of the glenoid demonstrating the planned implant positioning and surface contact mapping following osteophyte removal at the intended central implant contact area (red arrow). Image credit: Images generated by the authors using the ExactechGPS navigation system.

During the operation, a standard deltopectoral approach was utilized. The cephalic vein was identified and was retracted laterally with the deltoid, and deep self-retaining retractors were placed. The subscapularis and axillary nerve were palpated and protected. The subscapularis was then released, 1 cm medial to its insertion point, to allow for a cuff of tissue for later reattachment. Braided sutures were placed in a horizontal mattress fashion for lateral reattachment and now exposed the humeral head. There was significant full-thickness cartilage loss on both the humeral and glenoid sides. The long head of the biceps tendon was identified within its groove, and it had significant tendinopathy and flattening. It was brought out of its groove and transferred directly to the pectoralis and sutured circularly down using braided suture. The diseased intra-groove and intra-articular portions were removed. The supraspinatus and infraspinatus were protected with acromial retractors, and utilizing pre-op planning, a 20° retroversion guide, a proximal humeral cut was made. The humerus was then reamed by hand to size 13 for the stem and then broached to size 13 as well. A caliper was used, and then the 13 was left in place with an end cap. The glenoid was then exposed with a curved retractor posteriorly and a two-prong retractor anteriorly. The biceps stump, as well as any remaining labrum, was removed to fully expose the glenoid. The coracoid was then exposed using a two-prong retractor, and the G-Tracker (Exactech, Inc.) from the ExactechGPS navigation system (Exactech, Inc.) was secured using the short and long screws. Multiple registration points were made on the coracoid, coracoid neck, glenoid face, and glenoid neck. Intraoperative ExactechGPS navigation system (Exactech, Inc.) was used to verify accurate guide-pin placement and glenoid component positioning in real time (Figure 2).

Registration was accepted, and the central starting hole was drilled using a small and medium-sized reamer per plan. A medium-sized glenoid 8° posterior augment was planned. The central stem was again drilled for using the ExactechGPS navigation system (Exactech, Inc.). Three post holes were also drilled with appropriate rotation. A trial component was placed and demonstrated appropriate fit. The surgical site was irrigated thoroughly. Bone graft was packed into the stem of the glenoid component, which was then impacted into position. Excellent glenoid fixation was achieved. The G-Tracker (Exactech, Inc.) was subsequently removed along with the short and long screws.

Attention was then turned back to the humerus, where the trial in place was removed. Three drill holes were created for reattachment of the subscapularis, and sutures were passed back through using a suture passer. The site was irrigated once more, and the definitive size 13 stem was impacted into position. The 4.5 replication plate was placed, and utilizing the dials for appropriate coverage in angle, it was locked down with its break-off screw as well. The 47 x 18 mm head was trialed, and the shoulder was placed through a range of motion. Excellent stability was noted. The site was irrigated, and then the real head was impacted into position. The drill hole sutures were then reattached to bring our subscapularis back into position. This tendon was oversewn to the tendon utilizing the rim of tissue that we left using braided suture for dual fixation.

Following this, the shoulder was taken through the range of motion again. Forward elevation was 170°, external rotation was to 50° with no undue tension on the subscapularis. Irrigation was done a final time, hemostasis was confirmed, and then the surgical site was closed with Vicryl suture (Ethicon, Inc., Raritan, NJ) in the subcutaneous layer, and Monocryl (Ethicon, Inc.) for skin. The removal of the G-Tracker (Exactech, Inc.) and two screws was confirmed before closure. The shoulder was placed into a sling after a sterile compressive dressing, and the patient was awakened without difficulty and transferred from the bed to the recovery room in stable condition. No complications were encountered.

**Figure 3:**
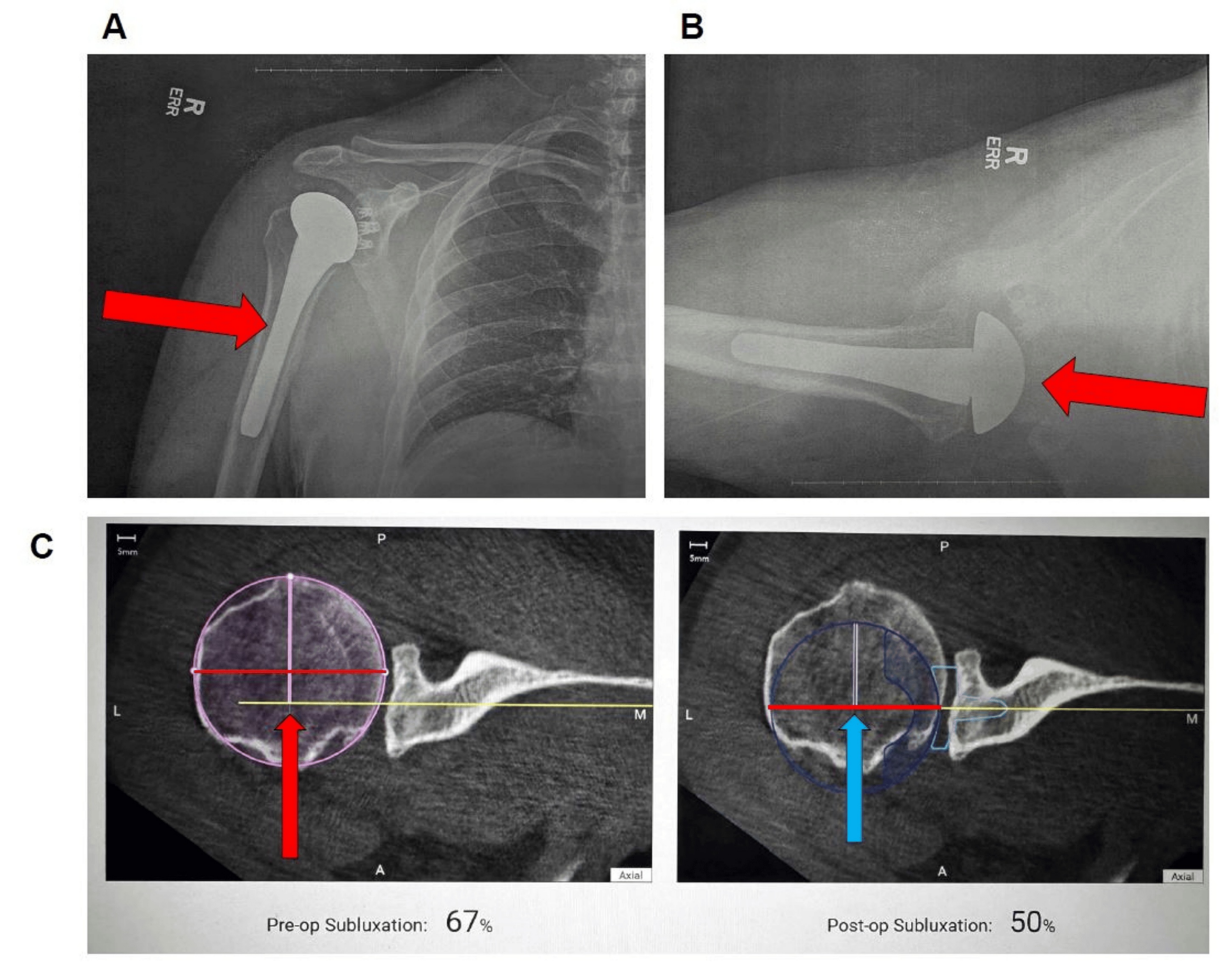
Postoperative imaging following an anatomic total shoulder arthroplasty performed using the ExactechGPS navigation system (Exactech, Inc., Gainesville, FL). (A) Anteroposterior radiograph demonstrating anatomic restoration of joint alignment and appropriate humeral head prosthesis positioning. (B) Axillary lateral view confirming concentric reduction and neutral version of the glenoid component without evidence of loosening or malposition. (C) Axial CT comparison demonstrating correction of posterior humeral head subluxation from 67% preoperatively (red arrow) to 50% postoperatively (blue arrow) following optimized glenoid version and implant placement.

The patient’s postoperative course was uncomplicated. He discontinued use of narcotic pain medication after two weeks and discontinued use of the sling one-month post-operation. He was progressing well in physical therapy, including isometrics and passive range of motion. At his three-month postoperative visit, the patient reported that he had returned to playing softball and experienced no issues with simple base hits or pitching, although he still had some difficulty throwing the ball properly. Postoperative imaging demonstrated anatomic restoration of joint alignment and appropriate component positioning (Figure 3).

Informed consent to write and publish this case was obtained verbally by the performing surgeon and the physical assistant.

## Discussion

aTSA remains the gold standard for patients with end-stage glenohumeral arthritis and an intact rotator cuff. However, its use in patients under 50 years of age continues to raise debate regarding implant longevity, revision risk, and postoperative expectations. In younger, high-demand individuals, balancing activity level with implant durability is critical for optimizing outcomes.

Recent high-level evidence supports the effectiveness of aTSA even in younger populations. In their 2022 systematic review and meta-analysis, Davies et al. evaluated 316 anatomic shoulder replacements in patients younger than 65 years. The authors reported a 94% implant survival rate at 10 years, decreasing to 81% at 20 years [4]. These findings demonstrate that while outcomes are generally excellent, revision risk increases over longer follow-up, particularly in patients under 50 due to higher activity levels and glenoid component wear.

Younger patients present unique challenges in arthroplasty decision-making. Their higher physical demands and longer life expectancy amplify cumulative stress across the glenoid-humeral interface, increasing risk for mechanical loosening and polyethylene wear. Additionally, degenerative deformities can distort the native glenoid version and bone stock, complicating component alignment. In this case, the use of the ExactechGPS navigation system (Exactech, Inc.) allowed precise intraoperative placement and version correction, mitigating one of the main technical risks associated with younger shoulders, malposition-induced eccentric loading [5]. This supports emerging evidence that navigation improves the accuracy of component orientation and may enhance long-term survival in high-demand individuals.

Our 48-year-old patient’s forward flexion (160°), external rotation (70°), and 5/5 cuff strength at three months are consistent with the early functional improvements reported in the literature. His radiographs demonstrated excellent alignment and fixation, and his rapid return to coaching and light sport activity parallels prior evidence that carefully selected younger patients can achieve near-normal shoulder function post-aTSA. Ongoing monitoring remains essential, as long-term studies indicate that mechanical loosening often emerges only after 10-15 years [5].

This case reinforces that aTSA, when combined with precise navigation techniques and appropriate patient selection, can yield excellent early outcomes in younger, high-demand individuals. Despite these advantages, national utilization remains relatively low, reflecting the novelty of the technology and limited longitudinal data. Continued multicenter follow-up of navigated aTSAs in this population is needed to determine whether enhanced alignment accuracy ultimately translates into superior implant survival.

## Conclusions

aTSA with the ExactechGPS navigation system (Exactech, Inc.) represents a valuable option for younger patients with end-stage glenohumeral osteoarthritis and an intact rotator cuff. Real-time three-dimensional navigation enhances the accuracy of glenoid component positioning, which may reduce technical complications and support early functional recovery. While this case demonstrates excellent short-term outcomes, long-term follow-up is necessary to determine whether improved intraoperative precision translates into sustained implant durability in high-demand individuals.

The patient reported high satisfaction both immediately after surgery and at one-year follow-up. He returned to his regular activities, including softball, conservative weightlifting, chopping wood, and coaching wrestling, without pain or functional limitation. He reported 0/10 pain and no postoperative complications.
